# Synergistic enhancement of cell death by triple combination therapy of docetaxel, ultrasound and microbubbles, and radiotherapy on PC3 a prostate cancer cell line

**DOI:** 10.1016/j.heliyon.2022.e10213

**Published:** 2022-08-15

**Authors:** Firas Almasri, Raffi Karshafian

**Affiliations:** a Department of Physics, Ryerson University, Toronto, Ontario, Canada; b Department of Mathematics and Natural Sciences, Gulf University for Science and Technology, Hawally, Kuwait; c Centre for Education Studies, University of Warwick, Coventry, UK; d Institute for Biomedical Engineering, Science and Technology (iBEST), A Partnership between Ryerson University and St. Michael's Hospital, Toronto, Ontario, Canada; e Keenan Research Centre for Biomedical Science of St. Michael's Hospital, Toronto, Ontario, Canada

**Keywords:** Chemotherapy, Ultrasound therapy, Sonoporation, Ultrasound and microbubble, Radiotherapy, Radio-enhancement

## Abstract

The application of ultrasound and microbubbles (USMB) has been shown to enhance both chemotherapy and radiotherapy. This study investigated the potential of triple combination therapy comprised of USMB, docetaxel (Taxotere: TXT) chemotherapy and XRT to enhance treatment efficacy. Prostate cancer (PC3) cells in suspension were treated with various combinations of USMB, chemotherapy and radiotherapy. Cells were treated with ultrasound and microbubbles (500 kHz pulse center frequency, 580 kPa peak negative pressure, 10 μs pulse duration, 60 s insonation time and 2% Definity microbubbles (v/v)), XRT (2 Gy), and Taxotere (TXT) at concentrations ranging from 0.001 to 0.1 nM for 5- and 120-minutes duration. Following treatment, cell viability was assessed using a clonogenic assay. Therapeutic efficiency of the combined treatments depended on chemotherapy and microbubble exposure conditions. Under the exposure conditions of the study, the triple combination therapy synergistically enhanced clonogenic cell death compared to single and double combination therapy. Cell viability of ∼2% was achieved with the triple combination therapy corresponding to ∼29, ∼37, and ∼38 folds decrease compared to XRT (57%), USMB (74%) and TXT (76%) alone conditions, respectively. In addition, the triple combination therapy decreased cell viability by ∼29, ∼19- and ∼11 folds compared to TXT_2hr_ + USMB (58%), TXT_2hr_ + XRT (37%), and USMB + XRT (22%), respectively.

The *in vivo* PC3 tumours showed that USMB significantly enhanced cell death through detection of apoptosis (TUNEL) with both TXT and TXT + XRT. The study demonstrated that the triple combination therapy can significantly enhance cell death in prostate cancer cells both *in vitro and in vivo* under relatively low chemotherapy and ionizing radiation doses.

## 1Introduction

Cancer treatment remains a challenge [1]. The combination of two or more therapeutic treatments has been shown to improve toxicity on tumour tissue and cells while minimizing side effects on normal tissues [2, 3]. Chemotherapy and radiotherapy, although clinically administered as individual therapies, their combination are often more effective at killing cancer cells [4, 5, 6]. Chemotherapy enhances radiotherapy by making cancer cells more susceptible to ionizing radiation bioeffects [4, 7, 8, 9]. Here, ultrasound in combination with microbubbles, which has intrinsic diagnostic and therapeutic attributes, is combined with both chemotherapy and radiotherapy. Microbubble agents, comprised of a shell-encapsulated gas-core microsphere, are generally, less than 5 μm allowing them to pass through the systemic circulation following peripheral venous administration. Several microbubble agents are approved for clinical use, including Definity (Lantheus Medical, Boston, MA), which is used in this study. These agents are clinically utilized to improve image quality, and detection of small blood vessels in tissues probed with ultrasound [10, 11]. Therapeutic ultrasound in combination with microbubbles are currently being investigated in targeted drug delivery applications. The ability of ultrasonically-stimulated microbubbles (USMB) to improve the delivery of drugs/genes to cells and tissues has been demonstrated, and novel applications are being developed in cancer, heart (myocardium), and neurology (blood-brain-barrier limited treatments), as well as in combination with chemotherapy or radiotherapy [12, 13, 14, 15, 16, 17].

This study investigated the efficacy of the triple combination therapy comprised of USMB, radiotherapy (XRT), and chemotherapy. The docetaxel (Taxotere: TXT) chemo-drug is used in this study. Docetaxel, a member of the taxane family, is used clinically for cancer treatment [18, 19]. As a result of the hydrophobic nature of both TXT and the semi-permeable membrane, the small docetaxel molecule can diffuse across the membrane. Studies have shown that increasing drug concentration and treatment duration lead to increased cell death, compliant with the diffusion mechanism [20, 21]. Furthermore, docetaxel is shown to cause cell cycle arrest in the G2/M phase by inhibiting depolymerization of microtubules and stabilizing tubulin, thus interfering with mitotic function and resulting in mitotic death [19, 22, 23]. Docetaxel enhances radiation therapy in part to its intrinsic ability to halt cells in the G2/M phase, where cells are generally radiosensitive [6]. The success of cancer therapy with chemotherapy is partly limited by their inability to localize treatments leading to undesired side effects, such as toxicity to healthy tissue. The application of ultrasound can be localized and combined with chemotherapy and synergistically enhance cancer cell death in [24, 25]. In addition, USMB can enhance the effect of XRT by activating the apoptosis pathway within cells [17, 26, 27]. USMB induced stresses on cancer, and endothelial cells triggered a membrane-activated mechanism associated with the ceramide-mediated cell death pathway, which might otherwise be triggered at high single doses of radiation, such as > 10 Gy [28]. Cells that produce ceramide are susceptible to apoptosis, and as a result, these cells are increasingly sensitive to radiotherapy. Furthermore, aside from the direct action of radiotherapy (DNA damage), XRT will also cause membrane damage through interactions with molecules within the cells, increasing ceramide levels and enhancing cell death [29, 30, 31].

This study aims to demonstrate the potential of a triple combination therapy comprised of USMB, chemotherapy (TXT), and radiotherapy (XRT) under relatively low exposure conditions of chemotherapy and radiotherapy. Ultrasound energy can be focused within the body non-invasively, and the application of USMB can be guided by ultrasound or other imaging modalities to selectively enhance the treatment of the tumour and spare surrounding normal tissues. The hypothesis guiding the study is that the triple combination therapy of TXT, USMB, and XRT can synergistically enhance cancer cell death compared to each treatment. The specific objectives are to determine the effect of the exposure conditions of chemotherapy by varying the TXT concentration and duration and the effect of MB concentration on the clonogenic viability of cancer cells using an *in vitro* prostate cancer cell line.

## 2Materials and methods

### 2.1Prostate cancer in vitro cell model

**Figure 1:**
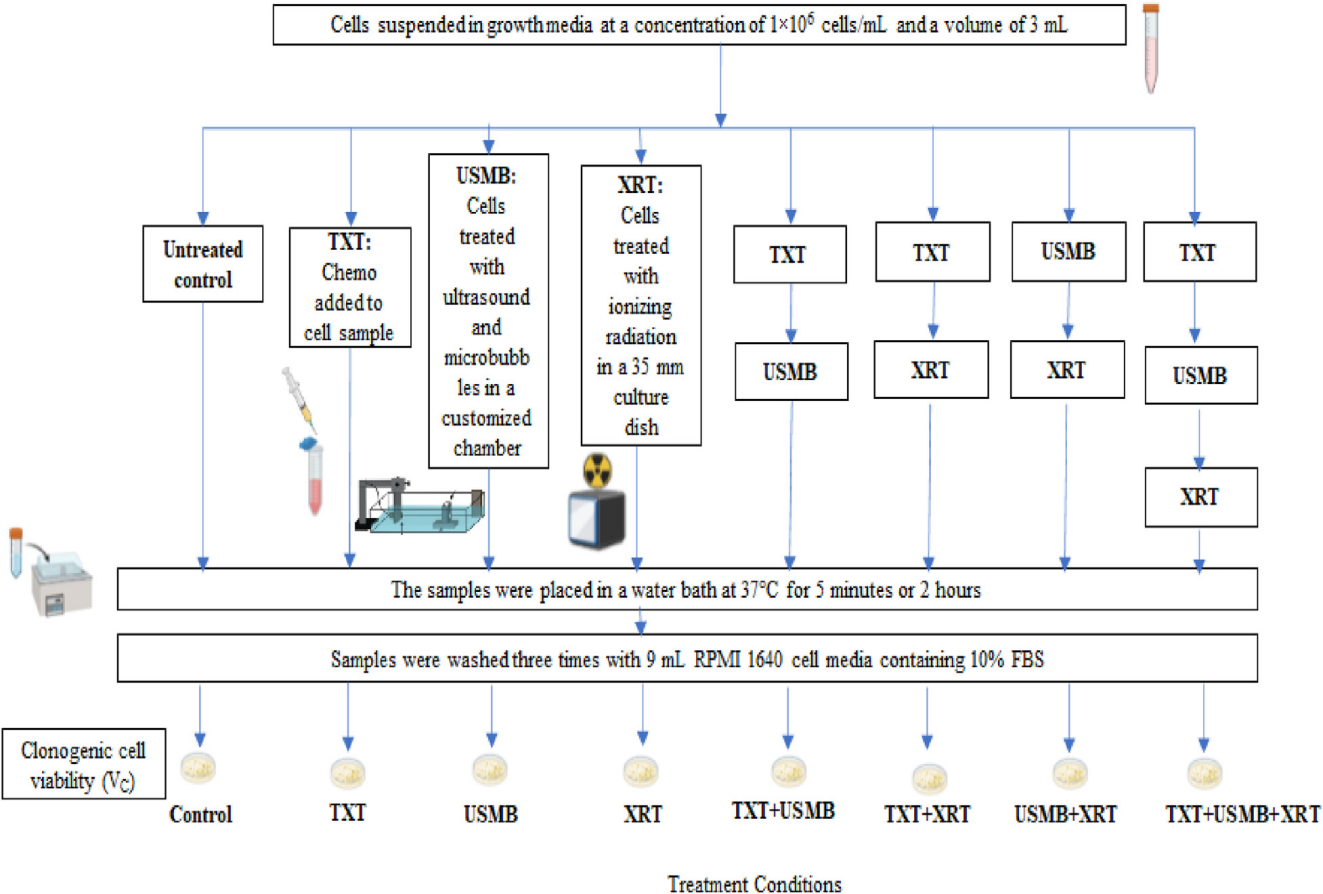
The overall experimental procedure and the different treatment conditions are represented here.

**Figure 2:**
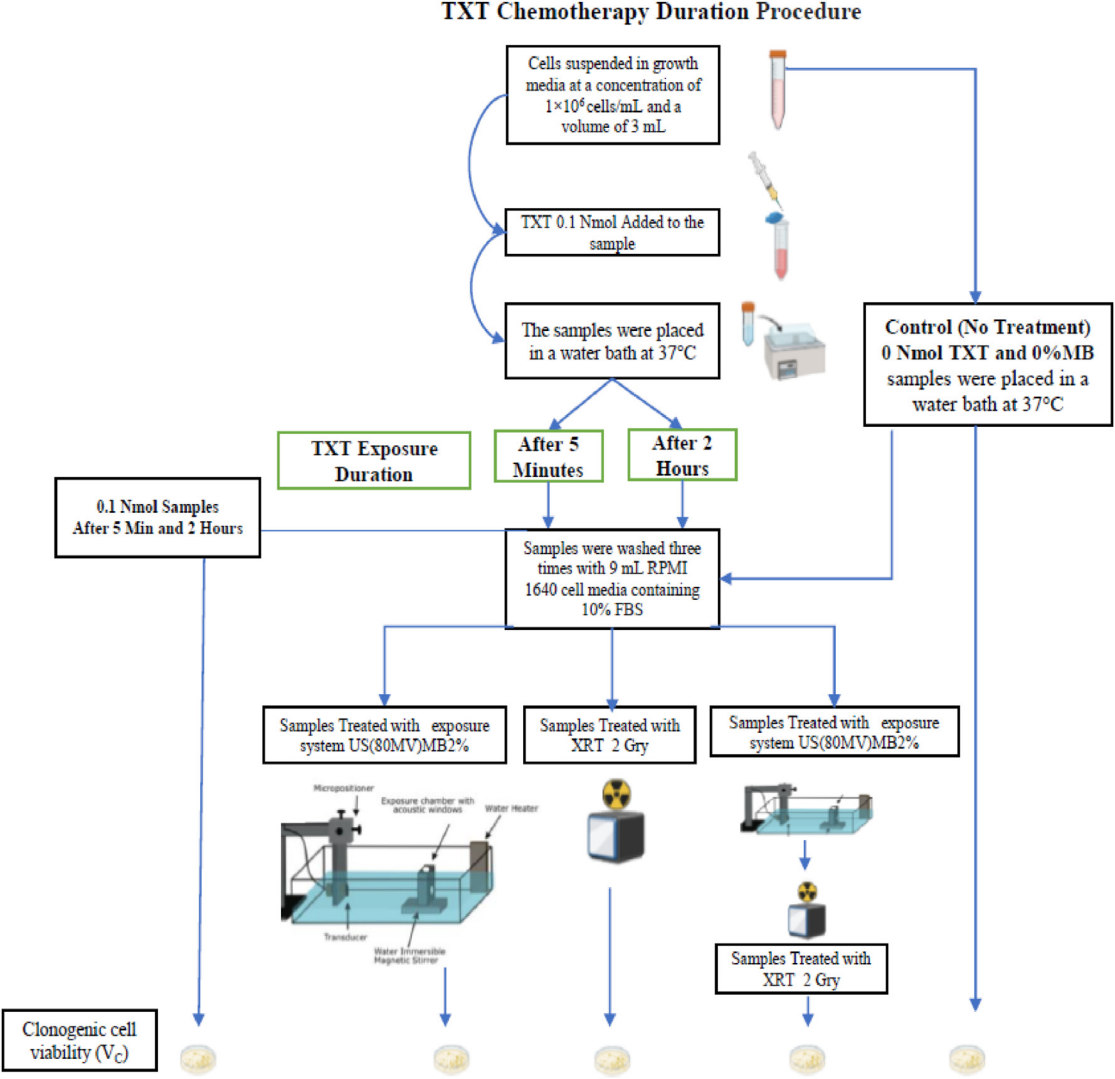
The overall experimental procedure and the different treatment conditions for TXT Chemotherapy Duration are represented here.

**Figure 3:**
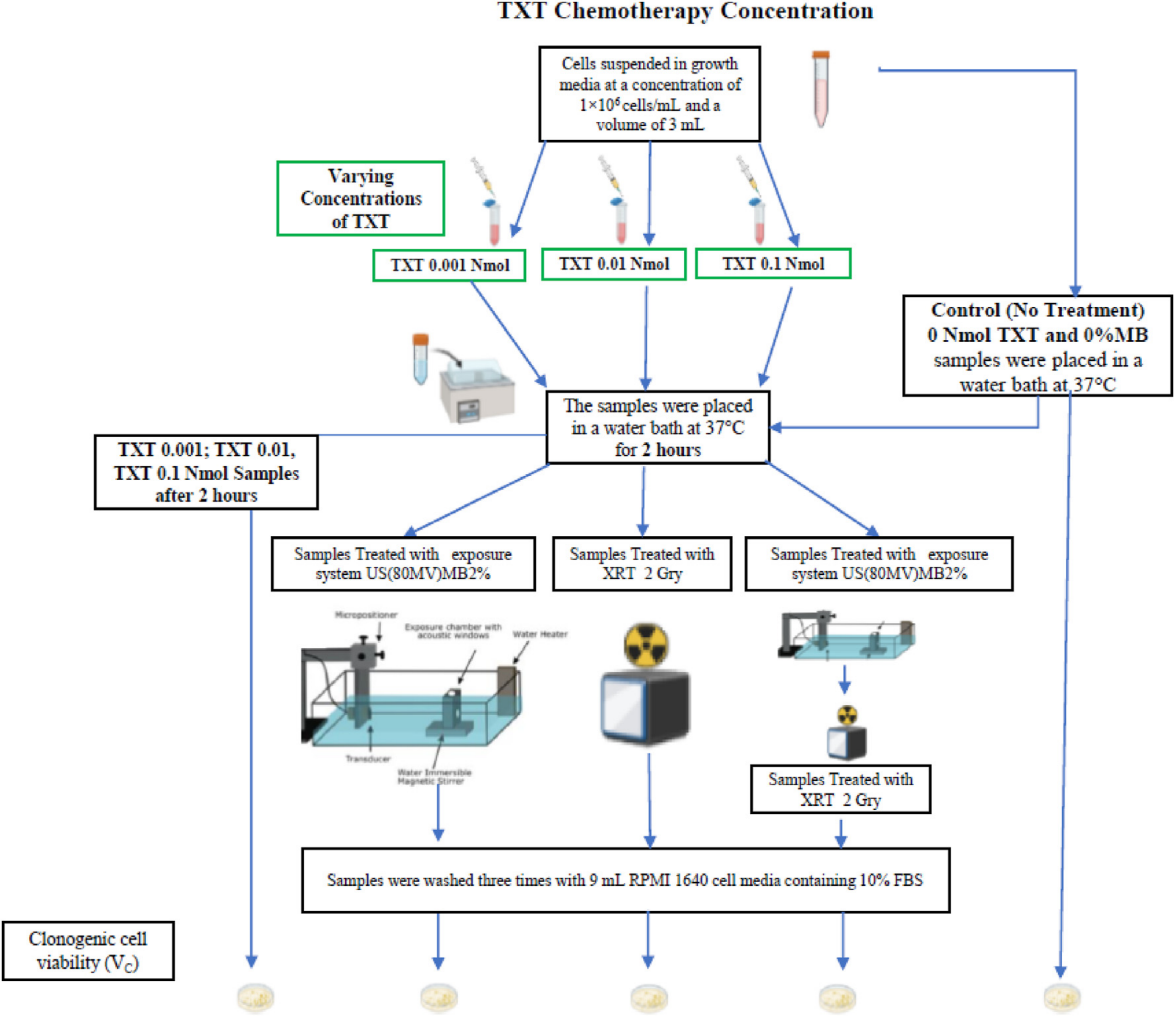
The overall experimental procedure and the different treatment conditions for TXT Chemotherapy Concentration are represented here.

**Figure 4:**
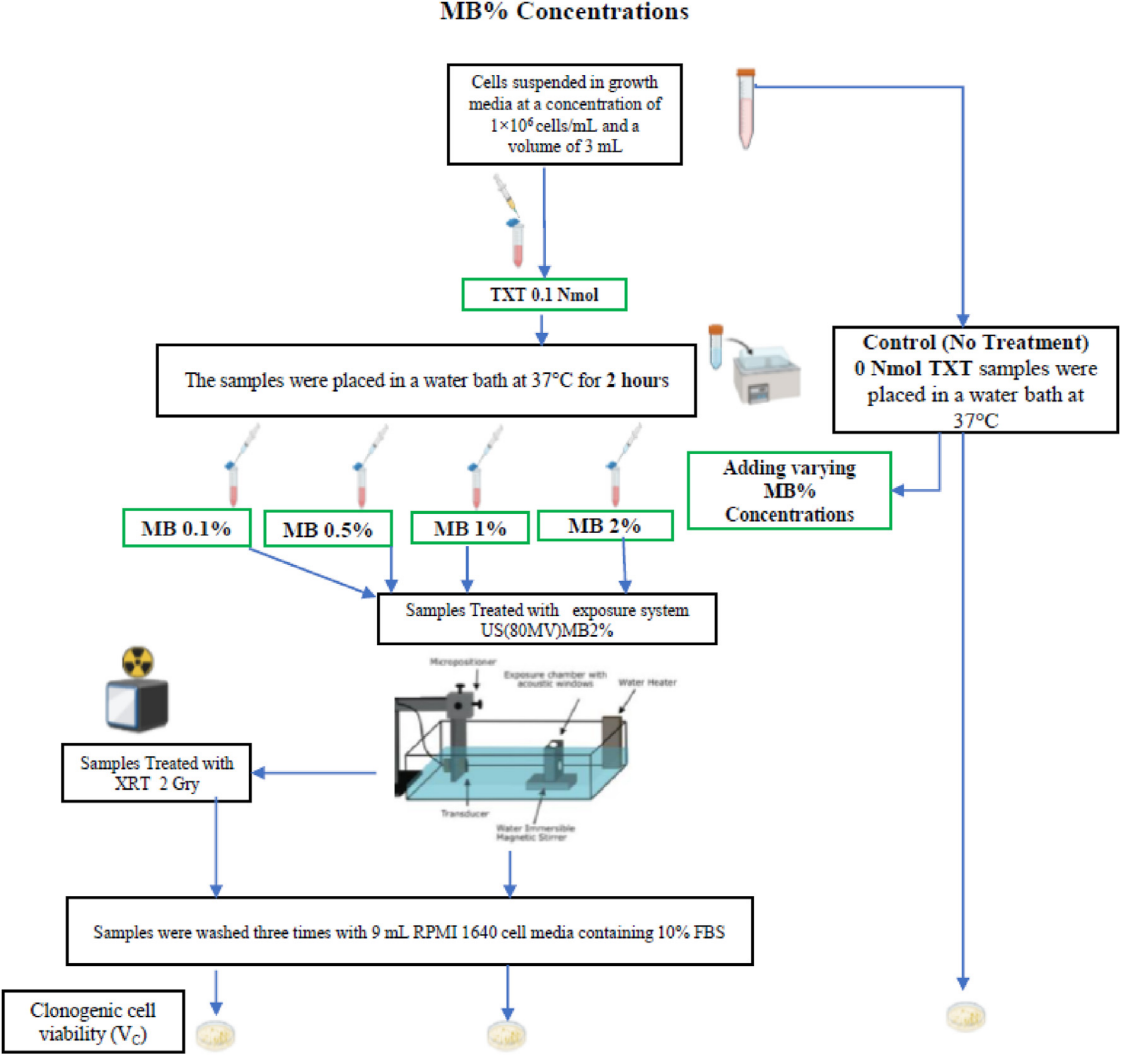
The overall experimental procedure and the different treatment conditions for Microbubble (MB) concentration are represented here.

Prostate cancer cells were treated with chemotherapy (TXT), radiotherapy (XRT), ultrasound, and microbubbles (USMB) using an *in vitro* cell suspension model. Human prostate cancer cell line (PC-3) (American Type Culture Collection, Manassas, VA, USA) were cultured using RPMI-1640 growth medium (Wisent, St Bruno, QC, Canada) supplemented with 1% penicillin/streptomycin (Gibco, Life Technologies, Burlington, ON, Canada) and 10% (volume concentration) fetal bovine serum (Thermo Scientific Hyclone, Logan, UT, USA). Cells were harvested during exponential growth using 0.05% Trypsin EDTA (GIBCO). PC3 were harvested from the cell culture flask, washed with RPMI-1640 growth medium, and resuspended at a concentration of 1 × 10^6^ cells/mL. *In vitro* experiments were conducted with cells suspended in growth media at a concentration of 1×10^6^ cells/mL and a volume of 3 mL. The overall experimental procedure and treatment conditions are shown in Figure 1. The details for each experiment are provided in Figure 2: TXT Chemotherapy Duration, Figure 3: TXT Chemotherapy Concentration, and Figure 4: Microbubble (MB) concentration.

### 2.2Prostate cancer in vivo cell model

Severe combined immunodeficient (SCID) male mice of ∼6 weeks of age and 20–30 g in mass (Charles River Laboratory International Inc. Canada) were injected sub-cutaneously with 50 μL (10^6^ cells) of suspended PC3 cells in their right hind legs; previously described [32]. The tumours, ∼6–9 mm in diameter, were treated with TXT, USMB, and XRTand sacrificed 24 h after treatment. Prior to therapy procedures, the animals were anaesthetized by IP injection of 0.1 mL volume comprised of ketamine 100 mg/kg, xylazine 5 mg/kg and acepromazine 1 mg/kg. The animals were treated with a volume of 100 μL dilution of TXT (MW 807.9 g/mol) at a concentration of 1.25 Dilution/Volume and administered through a tail-vein catheter and flushed with 100 μL saline solution. The USMB conditions were 500 kHz frequency for a duration of 2 min comprised of 50 ms sequence at 10 s sequence repetition period, and each sequence was composed of 32 μs pulse duration (16 cycles tone burst) and 3 kHz pulse repetition frequency. The XRT condition was an 8 Gy single-fraction dose at 160 kVp and 200 cGy/min dose rate (Faxitron Xray Corporation, Lincolnshire, IL, USA) [32]. Tumor samples were fixed for 24 h in 1.0% paraformaldehyde, and embedded in paraffin blocks. Segments were cut from distal-proximal ends of the tumor. Slices were then cytospinned at 2,000 × g and fixed for 30 min, followed by Tunel staining. The selection of apoptotic cells was done manually for the tunnel staining using the image, the positive tunnel area was divided by the total area to find the death percentage. The staining used at University Health Network (UHN) was a homemade kit, similar to the “Apoptag Kit” sold by EMD Millipore. The size of the labeling area correlated with the increase in treatments. Six different treatment conditions were investigated with four animals per condition; a total of 24 animals were used.

### 2.3Chemotherapy (TXT)

Docetaxel (Taxotere®; Aventis Pharmaceuticals, Inc.) was used in this study. *In vitro* cells samples were treated at doses of 0.001 nM (2 h), 0.01 nM (2 h), and 0.1 nM (5 min and 2 h) in a water bath at 37 °C. Following the treatment, cells were washed three times with 9 mL RPMI 1640 cell media containing 10% FBS; The overall experimental procedure and the different treatment conditions for TXT Chemotherapy Duration are represented in Figure 2, and the overall experimental procedure and the different treatment conditions for TXT Chemotherapy Concentration are represented in Figure 3.

### 2.4Radiotherapy (XRT)

A single fraction ionizing radiation at 160 kVp X-rays at 200 cGy/min dose rate (Faxitron X-ray Corporation, Lincolnshire, IL, USA) was used. *In vitro* cells in an RPMI growth medium were placed in 35 mm culture dishes and exposed to a 2 Gy XRT dose.

### 2.5Ultrasound and Microbubble (USMB)

The ultrasound exposure system consisted of a single element transducer 500 kHz center frequency (IL0509HP, Valpey Fisher Inc., Hopkinton, MA), a waveform generator (AWG520, Tektronix Inc, Beaverton, OR), and a power amplifier (RPR4000, Ritec Inc, Warwick, RI). The microbubble agent used in these experiments was Definity® (Lantheus Medical Imaging, Inc., North Billerica, MA, USA), which is clinically approved for ultrasound imaging. The microbubbles were prepared by activating the Definity® vial using a Vialmix® (Lantheus Medical Imaging, Inc., North Billerica, MA, USA) for 45 s. The microbubbles were at room temperature during activation. The overall experimental procedure and the different treatment conditions for Microbubble (MB) concentration are represented in Figure 4.

### 2.6Experimental conditions

*In vitro* cells were placed into an acoustic chamber that contained an immersible magnetic stirrer and exposed to ultrasound and microbubbles [33]. Cells were exposed to 500 kHz pulse center frequency, 32 μs pulse duration, and 3 kHz pulse repetition frequency (PRF) at 580 kPa peak negative pressure amplitude for 60 s in the presence of microbubbles. These parameters were chosen based on previous experiments [33]. The microbubble concentration was varied from 0-to-2% volume concentration (0, 0.1, 0.5, 1 and 2%). Cells were exposed to ultrasound pulses immediately following the addition of microbubbles. In the combined treatment, docetaxel was added immediately before exposing the cells to USMB. The chemotherapy duration, chemotherapy dose, microbubble concentrations, and order of the treatments were varied in this study. Each experimental condition involving the various combinations of TXT, USMB, and XRT was performed in triplicates. The untreated control samples were placed in a water bath at 37 °C prior to clonogenic assay (as shown Figure 1). TXT was placed inside the sample for either 5 min or 2 h and was washed 3 times with media (RPMI) following the completion of each treatment. During the treatment of TXT + USMB samples, the TXT was added prior to USMB exposure. When treating samples with XRT in combination with USMB or TXT, samples were treated with XRT 2 min post-exposure to USMB or TXT. Thus, the experimental condition for combining all three treatment types was achieved by simultaneously treating samples with USMB and TXT, then with XRT 2 min post TXT + USMB.

### 2.7Clonogenic assay

Following the treatment, cell viability was assessed through their ability to proliferate and form a colony. Cells (100 cells/mL at 1 mL volume) were plated in 50 mm Petri dishes containing 4 mL cell media culture and incubated for 14 days. The cells were stained with Methylene blue (1% w/v, VWR International, Ontario, Canada) and counted using a microscope. Each experiment was repeated three times with five samples per condition.

Clonogenic cell viability (V_C_) represents the percentage of cells that were able to form a cell colony. The V_C_ is calculated using:

$$V_{C}=\;\frac{Colonies\;counted}{Cells\;seeded\;\times \;PE}\times 100\%$$

Where the “Colonies counted” represents the number of colonies formed, “Cells seeded” represents the number of seeded cells, and PE represents the plating efficiency ratio (the number of colonies counted divided by the number of seeded cells of the untreated control sample).

The viability ratio (V_R_) was used to compare the response within each treatment group. That is, the V_R_ of a sample treated with TXT + USMB is calculated by dividing the V_C_ of TXT + USMB by the V_C_ of USMB alone; the V_R_ of cells treated with TXT + XRT is calculated by dividing the V_C_ of TXT + XRT by the V_C_ of XRT alone, and the V_R_ of cells treated with TXT + USMB + XRT is calculated by dividing the V_C_ of TXT + USMB + XRT by the V_C_ of USMB + XRT. The V_R_ is used to make such data comparable across the treatment. The data are presented as mean ± SD.

### 2.8Statistical analysis

The mean and standard deviation of treatment effect as measured by clonogenic cell viability are presented. T-test and 2-way analysis of variance was used to examine statistical significance between treatment conditions. P-values< 0.05 were considered significant. Cell survival assays were done in triplicates, and conditions were repeated at least five times (i.e., three colony culture dishes per sample and five samples per condition).

**Figure 5:**
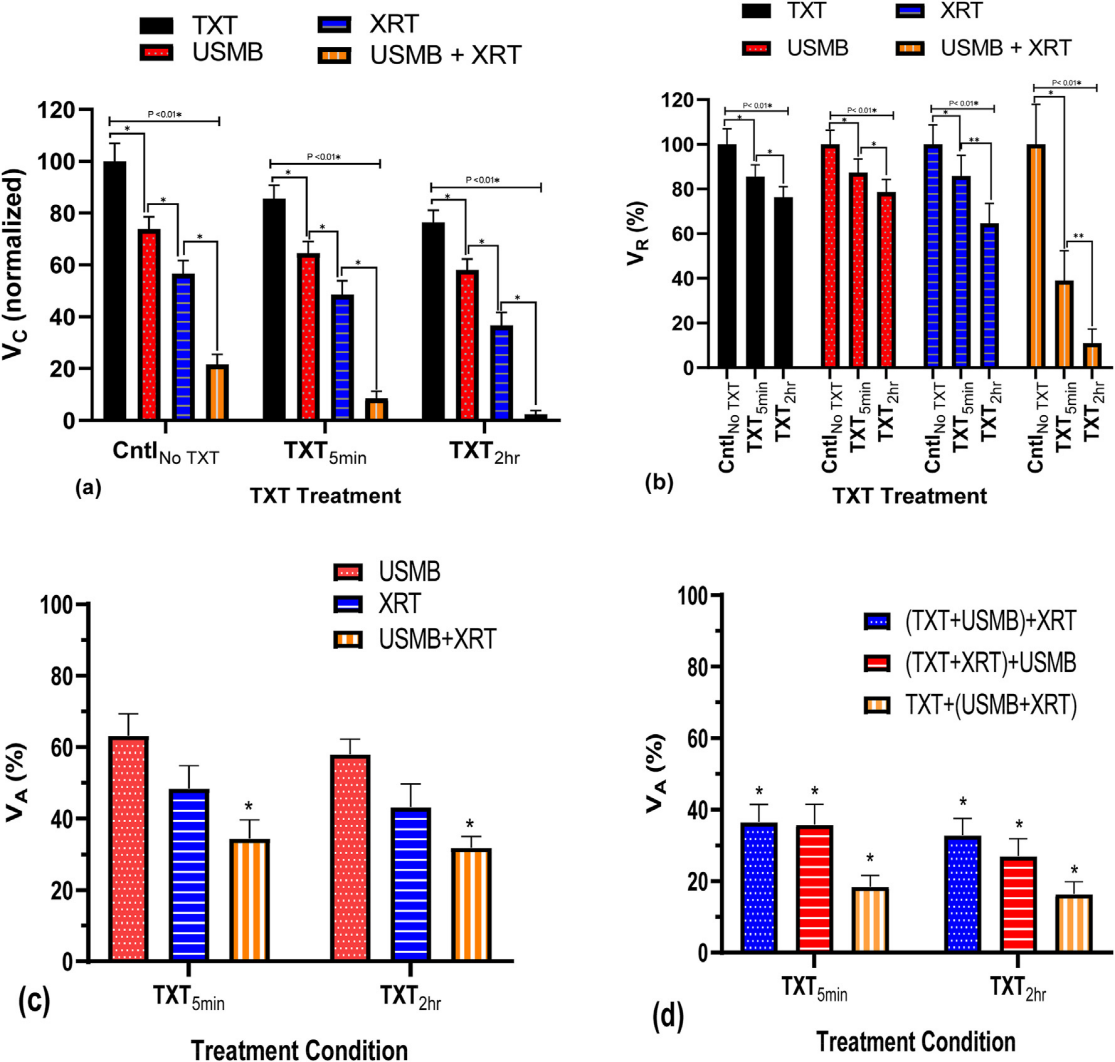
(a) PC3 clonogenic viability (V_C_) exposed to 0.1 nM TXT at two different treatment exposure times (5 min and 2 h); cell viability was normalized to the untreated control. USMB is fixed at 0.5 MHz frequency pulses with 580 kPa negative peak pressure and 2% (v/v) microbubbles, and a 160 kVp 2Gy single radiation dose, and their combinations. (b) The viability ratio (V_R_) normalized to zero nM TXT for each combined treatment, where PC3 cells been exposed to 0.1 nM TXT at two different treatment exposure times (5 min and 2 h); cell viability was normalized to the untreated control. USMB is fixed at 0.5 MHz frequency pulses with 580 kPa negative peak pressure and 2% (v/v) microbubbles, and a 160 kVp 2 Gy single radiation dose (∗ P < 0.05). (c) The calculated additive cell viability (V_A_) of TXT, USMB, and XRT for 5 min and 2 h TXT treatment duration based on three independent experimental treatments. (d) The calculated additive effect (V_A_) of different permutations of TXT + USMB + XRT 5 min and 2 h exposure time based on two independent experimental treatments. These samples were centrifuged, removing TXT within the sample before irradiation and plating in dishes. N = 24 from 4 independent experiments. The asterisks in (c) and (d) identify the treatments that are synergistic; that is, when V_C_ is statistically lower than V_A_ (∗ P < 0.05).

**Figure 6:**
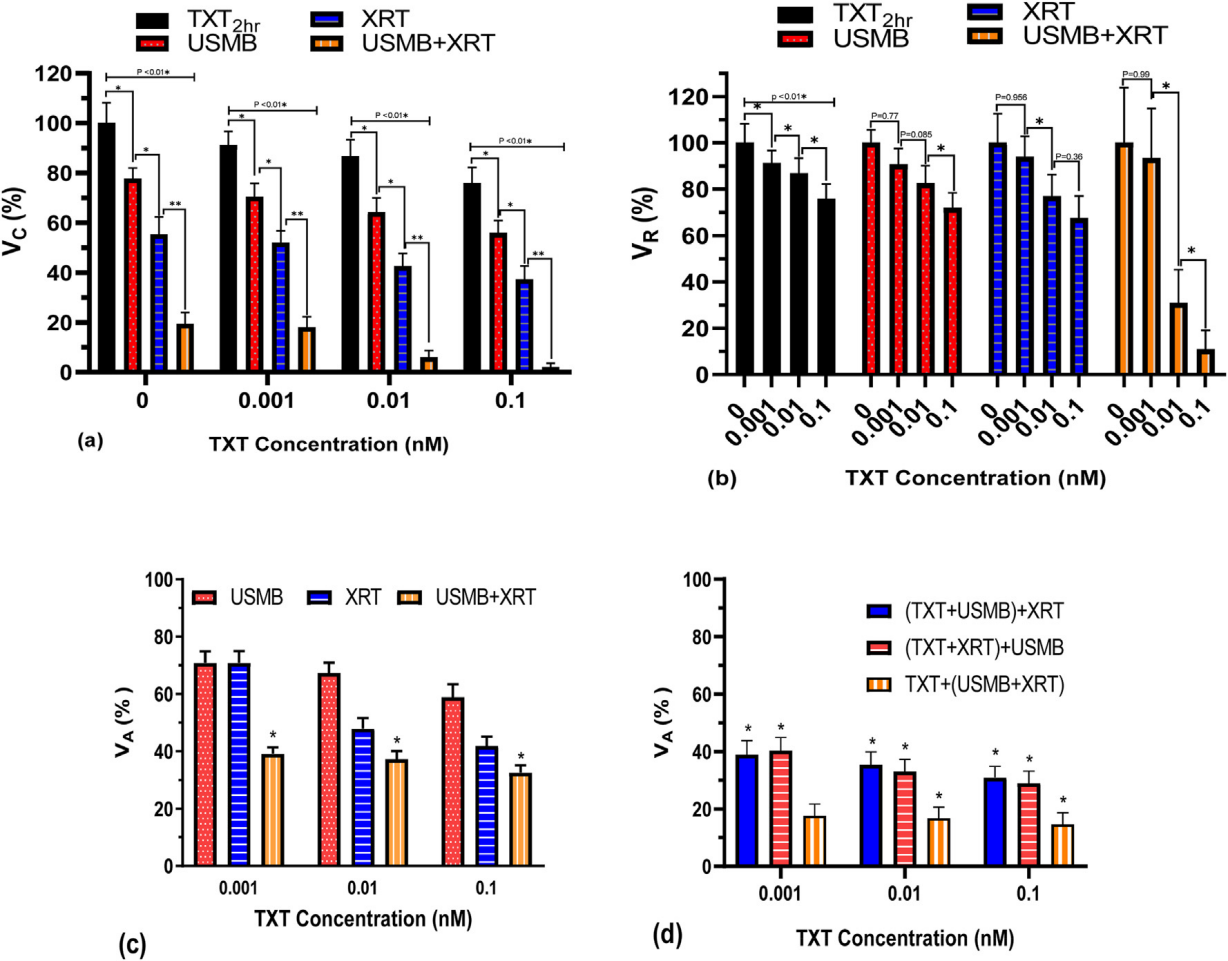
(a) Clonogenic viability (V_C_) of PC3 cells exposed to different TXT concentrations (0, 0.001, 0.01, 0.1 nM) for 2 h treatment duration at Pneg = 580 kPa and 2% (v/v) microbubble concentration, and 160 kVp 2 Gy single radiation dose, and various treatment modality combinations; cell viability was normalized to the untreated control. (b) The viability ratio (V_R_) for the corresponding treatments normalized to each combined treatment. (∗ P < 0.05). (c) The calculated additive effect (V_A_) of TXT, USMB and XRT for varying TXT concentrations. (d) The calculated additive effect (V_A_) of different permutations of TXT + USMB_XRT for varying TXT concentrations. N = 15 from three independent experiments. The asterisks in (c) and (d) identify the treatments that are synergistic; that is when V_C_ is statistically lower than V_A_ (∗ P < 0.05).

**Figure 7:**
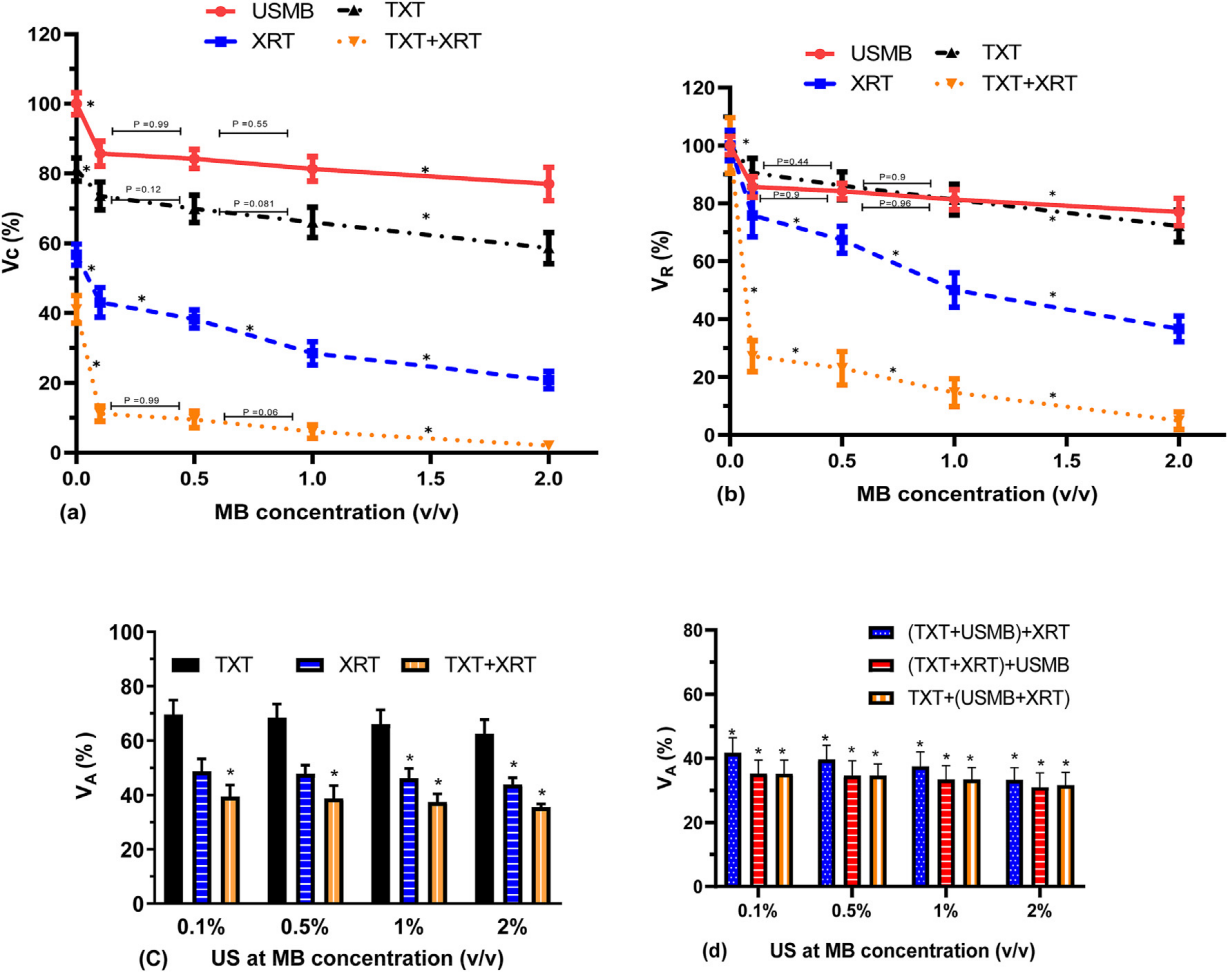
(a) Clonogenic viability (V_C_) of PC3 cells exposed to different MB concentrations (0, 0.1, 0.5, 1.0 and 2.0% volume concentration) at Pneg = 580 kPa in combination with TXT (0.1 nM and 2 h duration) and 160 kVp 2 Gy single radiation dose, and various treatment modality combinations; cell viability was normalized to the untreated control. (b) The viability ratio (V_R_) for the corresponding treatments normalized to each combined treatment, were PC3 cells exposed to different MB concentrations (0, 0.1, 0.5, 1.0 and 2.0% volume concentration) at Pneg = 580 kPa in combination with TXT (0.1 nM and 2 h duration) and 160 kVp 2 Gy single radiation dose, and various treatment modality combinations (∗ P < 0.05). (c) Additive cell viability (V_A_) of TXT, USMB and XRT for varying MB concentrations based on three independent treatment modalities. (d) Additive cell viability (V_A_) of different permutations of TXT + USMB + XRT for varying concentrations of MB based on two independent treatment modalities. N = 18 from three independent experiments. The asterisks in (c) and (d) identify the treatments that are synergistic; that is, when V_C_ is statistically lower than V_A_ (∗ P < 0.05).

The synergism of the combined treatment was assessed using the Bliss independence model [34]. The combined treatment was considered synergistic when the experimentally assessed cell viability (V_C_) was statistically lower than the expected additive effect calculated value (V_A_). The V_A_ calculation is based on two approaches. The first approach is based on three independent treatment modalities; TXT, USMB, and XRT. The V_A_ is calculated by the following equation: V_A=_ V_C1_ × V_C2_ × V_C3_ where V_C1,_ V_C2,_ and V_C3_ represent cell viabilities of single treatments representing TXT, USMB and XRT. The analysis based on this approach are shown in Figures 5c, 6c and 7c. The second approach is based on two independent treatments, where a treatment is assumed to consist of two single treatments. For example, one treatment can be TXT and the second treatment can be USMB + XRT (for these calculations, USMB + XRT represents a single experimental treatment). Here, the V_A_ is calculated by the following equation: V_A=_ V_C1_×V_C2,_ where V_C1_ and V_C2_ represent clonogenic cell viabilities. These analyses are shown in Figures 5d, 6d, and 7d.

## 3Results

The triple combination treatment of TXT + USMB + XRT induced a synergistic effect on cell death compared to each single treatment modality and to each of the double combination treatment modalities. The triple combination therapy of TXT, USMB, and XRT significantly improved cell death compared to each treatment alone. Cell viability (V_C_) of the triple combination treatment (TXT_2hr_ + XRT + USMB = 2%) in PC3 cells decreased by ∼28, ∼37 and ∼38 folds compared to XRT alone (57%), USMB (74%) and TXT (76%), respectively. In addition, the triple combination therapy significantly decreased cell viability by ∼29, ∼19- and ∼11 folds compared to TXT_2hr_ + USMB (58%), TXT_2hr_ + XRT (37%), and USMB + XRT (22%), respectively. The therapeutic efficiency of the combined treatment depended on chemotherapy duration and concentration, as well as MB concentration.

### 3.1TXT chemotherapy duration

Clonogenic viability of cells (V_C_: Cell viability normalized with respect to the untreated control) treated with TXT for 5 min and 2 h duration at 0.1 nM concentration, USMB (2% MB concentration), XRT (2 Gy) and their combinations are shown in Figure 5a and Table 1. The viability ratio (V_R_: Cell viability normalized with respect to each treatment condition TXT, USMB, XRT and USMB + XRT) is shown in Figure 5b. The therapeutic effect (synergism) of all three treatments (TXT, USMB and XRT) was calculated using the Bliss independence criterion. Figure 5c shows the additive cell viability (V_A_) based on three independent treatment modalities, and Figure 5d shows the additive cell viability (V_A_) based on two independent treatment modalities of various combinations of TXT, USMB, and XRT. Each treatment alone decreased cell viability. The lowest cell viability was achieved with the combined treatment (TXT + USMB + XRT) at 2 h TXT exposure duration. The effect of the combined (TXT_2hr_ + USMB + XRT) treatment resulted in cell viability of ∼2%, ∼4-folds less than that of the shorter duration (TXT_5min_ + USMB + XRT). The cell viability of the combined treatment (TXT_2hr_ + USMB + XRT) resulted in a ∼9-folds and ∼3-folds decrease at 0.1 nM TXT concentration compared to 0.001 nM and 0.01 nM, respectively. In addition, cell viability treated with TXT + USMB + XRT at 2 h (V_C_ = 2%) and 5 min (V_C_ = 8%) is statistically lower compared to USMB + XRT (V_C_ = 22%) (Figure 5a). The combination of TXT at 2 h and 5 min with USMB + XRT synergistically reduced V_C_ by ∼11 and ∼4 folds, respectively, in relation to USMB + XRT. The viability ratio (V_R_) shows the significant dependence of TXT exposure duration in the combined treatment of TXT + USMB + XRT (Figure 5b). The combined treatment of TXT + USMB + XRT synergistically stimulated cell death at both chemotherapy durations (Figure 5c and 5d). The calculated additive cell viabilities of TXT + USMB + XRT of V_A_ = 32% and V_A_ = 35% for 2 h and 5 min, respectively, based on three independent treatments (Figure 5c), were statistically different compared to experimental cell viabilities (V_C_). Furthermore, the combined treatment of TXT + USMB + XRT was synergistic even under consideration of two-combined treatment modalities (Figure 5d). The different permutations of TXT + USMB + XRT at 2 h and 5 min were investigated to determine a possible synergistic mechanism by comparing the experimental cell viability to the calculated additive values. The additive calculated cell viabilities for (TXT + USMB)+XRT (V_A_ is 16% and 18%), (TXT + XRT)+USMB (V_A_ is 33% and 37%) and TXT+(USMB + XRT) (V_A_ is 27% and 36%) for 2 h and 5 min, respectively, were statistically lower compared to clonogenic cell viability (Figure 5d).

**Table 1:** Cell viability (V_C_) normalized with respect to the untreated control treated with TXT for 5 min and 2 h duration at 0.1 nM concentration, USMB (2% MB concentration), XRT (2 Gy) and their combinations.

TXT Time	TXT 0.1 nmol			TXT 0.1 nmol + US(80MV)MB2%			TXT 0.1 nmol + XRT 2 Gy			TXT 0.1 nmol + US(80MV)MB2% + XRT 2 Gy		
	Mean	SD	N	Mean	SD	N	Mean	SD	N	Mean	SD	N
Cntl_No TXT_	100.00	6.92	24	73.87	4.68	24	56.63	4.98	24	21.57	3.88	24
TXT_5min_	85.50	5.24	24	64.55	4.45	24	48.59	5.23	24	8.43	2.86	24
TXT_2hr_	76.30	4.80	24	58.02	4.19	24	36.61	5.06	24	2.36	1.39	24

Within each treatment group (Cntl_No TXT_, TXT_5min_ and TXT_2hr_), a statistically significant difference was observed between of all conditions (P < 0.05). USMB improved the therapeutic response of XRT in the absence of TXT. Cell viability with USMB + XRT (V_C_ = 22%) was significantly lower compared to USMB and XRT alone (V_C_ = 76% and 57%, respectively); Figure 5a, corresponding to a ∼3 fold decrease with USMB + XRT compared to XRT alone. The USMB + XRT combined treatment induced a synergistic effect compared to the calculated cell viability (V_A_ = 42%). TXT improved the therapeutic outcome of both USMB and XRT with higher enhancement in cell death at the longer TXT treatment duration. Cell viability decreased by 20% in the TXT_2hr_ + XRT (V_C_ = 37%) treatment whereas by 8% in the TXT_5min_ + XRT (V_C_ = 49%) compared to XRT (V_C_ = 57%) alone. Comparable decreases in cell viability (V_C_) were observed within TXT + USMB and TXT alone treatments. TXT alone induced 25% and 15% decrease in V_C_ relative to the untreated control, at 2 h and 5 min TXT exposure durations, respectively. Furthermore, an average decrease of ∼20% in cell viability was observed when treating cells with TXT + USMB compared to TXT alone for both durations (Figure 5a). However, at the concentration (0.1 nM) and treatment durations (5 min and 2 h), the TXT + XRT and TXT + USMB combined-treatments were additive. The additive calculated cell viabilities (V_A_ = 43% and 56%, respectively) for the 2h chemotherapy treatment was statistically comparable to experimentally measured cell viabilities (V_C_ = 37% and 58%, respectively), indicating additive effects. For the short treatment time (5 min), the additive calculated cell viability (V_A_ = 48% and 63% respectively) was comparable to the experimental cell viability value (V_C_ = 49% and 65% respectively) for each treatment.

### 3.2TXT chemotherapy concentration

The clonogenic viability of cells (V_C_) exposed to varying concentrations of TXT (0, 0.001, 0.01 and 0.1 nM) for 2 h duration, USMB (2% MB concentration), XRT (2 Gy) and their combinations are shown in Figure 6a (Table 2), and the corresponding viability ratio (V_R_) is shown in Figure 6b. The additive cell viabilities (V_A_) based on three independent treatment modalities is shown in Figure 6c. The V_A_ based on two independent treatment modalities of various combinations of TXT, USMB, and XRT is shown in Figure 6d. Generally, cell viability decreased with TXT concentration when treated with TXT alone or combined with USMB and XRT. In the TXT + USMB and TXT + XRT treatment conditions, V_C_ decreased by ∼6–9% with each fold increase in TXT concentration (Figure 6a). Under these conditions, the combined treatments of TXT + USMB and TXT + XRT induced an additive effect (Figure 6c). The additive calculated cell viabilities (V_A_ = 42%, 48%, and 70%, respectively) pertaining to the TXT + XRT treatment were statistically comparable to the experimentally measured cell viabilities (V_C_ = 37%, 43% and 52%, respectively), indicating additive effects. Similarly, the TXT + USMB treatment also resulted in cell viabilities (V_C_ = 56%, 64%, and 70%, respectively) that were analogous to calculated values (V_A_ = 57%, 65%, and 68%, respectively).

**Table 2:** Cell viability (V_C_) normalized with respect to the untreated control exposed to varying concentrations of TXT (0, 0.001, 0.01 and 0.1 nM) for 2 h duration, USMB (2% MB concentration), XRT (2 Gy) and their combinations.

TXT Dose	TXT_2hr_			TXT_2hr_ + US(80MV)MB2%			TXT_2hr_ + XRT-2 Gy			TXT_2hr_ + US(80MV)MB2% + XRT		
	Mean	SD	N	Mean	SD	N	Mean	SD	N	Mean	SD	N
0 nmol	100.00	8.12	24	77.72	4.34	24	55.32	6.94	24	19.35	4.62	24
0.001 nmol	91.17	5.48	24	70.45	5.37	24	51.95	4.89	24	18.05	4.18	24
0.01 nmol	86.69	6.72	24	64.15	5.89	24	42.53	5.19	24	5.97	2.77	24
0.1 nmol	75.78	6.38	24	55.91	5.05	24	37.34	5.30	24	2.08	1.62	24

Cell viability (V_C_) was significantly lower in the combined treatment of TXT + USMB + XRT compared to single and double-treatment combinations (Figure 6c and 6d). In the TXT + USMB + XRT treatment, the lowest V_C_ was achieved at 0.1 nM, the highest concentration used in this study. A statistically significant difference in V_C_ was achieved at 0.01 and 0.1 nM TXT concentration, whereas minimal reduction in cell viability was observed at 0.001 nM TXT concentration (Figure 6b). The combined treatment of TXT + USMB + XRT was synergistic at all TXT concentrations based on the calculations of three independent treatment modalities (Figure 6c). Cell viability (V_C_ = 2%, 8%, 18% for 0.1, 0.01 and 0.001 nM, respectively) was significantly lower compared to the additive calculated values (V_A_ = 31%, 36%, 38% for 0.1, 0.01 and 0.001 nM, respectively). Whereas, based on synergism analysis of two independent treatment modalities, the combined treatment of TXT + USMB + XRT, except when compared with V_A_ at 0.001 nM based on TXT + (USMB + XRT) analysis. This suggests that the synergism in the combined treatment of TXT + USMB + XRT at 0.001 nM is associated with the synergistic effect of USMB + XRT.

### 3.3Microbubble (MB) concentration

Clonogenic cell viability (V_C_) exposed to varying concentration of MB (0%, 0.1%, 0.5%, 1.0% and 2.0% volume concentration), 0.1 nM TXT for 2 h duration, XRT (2 Gy) and their combinations are shown in Figure 7a (Table 3), and the corresponding viability ratio (V_R_) in Figure 7b. Additive cell viability (V_A_) based on three- and two-independent treatment modalities are shown in Figure 7c and 7d, respectively. Generally, V_C_ decreased with increasing MB concentration at all treatment conditions. V_C_ is statistically different between the different treatment conditions at each MB concentration (Figure 7a). In the TXT + USMB condition, the treatment is additive at all MB concentrations, whereas in the USMB + XRT condition, the combined treatment is synergistic at MB concentrations of 1% and 2% (Figure 7c). In the absence of microbubbles, V_C_ with the combined treatment of TXT + US + XRT was significantly lower compared to each treatment modality, where the combined treatment was additive. The addition of microbubbles induced a significant effect on cell viability. Cell viability in the TXT + USMB + XRT condition decreased by ∼4 and ∼18 folds at MB concentrations of 0.1% and 2% v/v (V_C_ = 12% and 2%, respectively). The combined treatment of TXT + USMB + XRT was synergistic based on three- and two-independent treatment modality analysis at MB concentrations of 0.1%–2% (Figure 7c and 7d).

**Table 3:** Cell viability (V_C_) normalized with respect to the untreated control exposed to varying concentration of MB (0%, 0.1%, 0.5%, 1.0% and 2.0% volume concentration), 0.1 nM TXT for 2 h duration, XRT (2 Gy) and their combinations.

MB concentration	US(80MV)MB			XRT-2 Gy + USMB(80MV)			USMB(80MV) + TXT_2hr_			US(80MV)MB + TXT_2hr_ (0.1 nmol) + XRT-2 Gy		
	Mean	SD	N	Mean	SD	N	Mean	SD	N	Mean	SD	N
USMB 0%	100.00	5.23	24	56.73	4.19	24	81.15	3.38	24	41.07	3.97	24
USMB 0.1 %	85.72	4.68	24	43.05	4.24	24	73.54	3.98	24	11.19	3.02	24
USMB 0.5 %	84.18	2.73	24	38.26	4.04	24	69.85	3.93	24	9.48	2.39	24
USMB 1 %	81.31	3.50	24	28.45	3.37	24	65.99	4.35	24	6.01	2.35	24
USMB 2 %	77.01	4.77	24	20.78	3.18	24	58.60	4.47	24	2.04	1.25	24

### 3.4In vivo TUNEL stain

**Figure 8:**
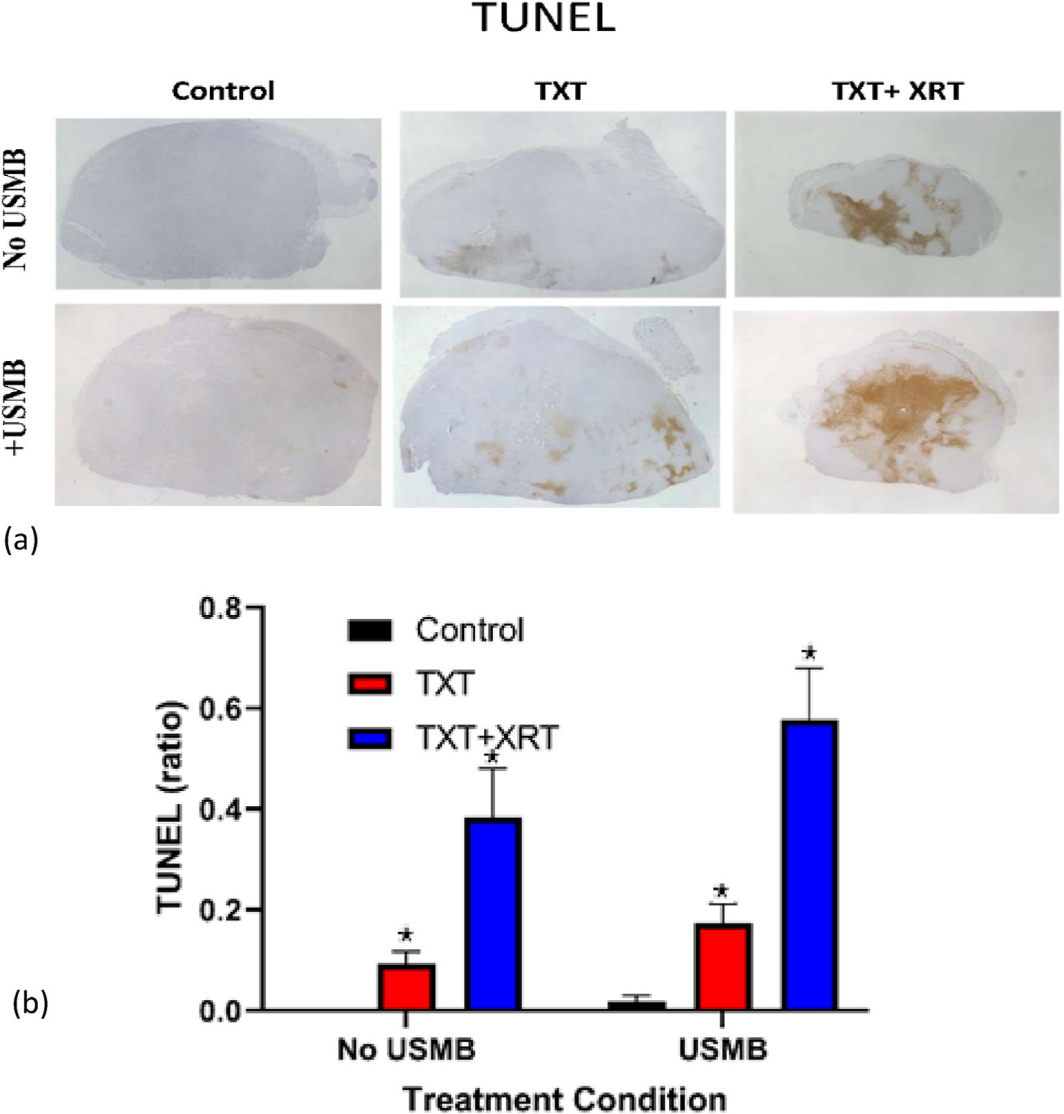
(a) TUNEL assay of histological tumour sections at six treatment conditions: Untreated control, docetaxel alone (TXT), docetaxel and radiotherapy (TXT + XRT), ultrasound-microbubble alone (USMB), ultrasound-microbubbles and docetaxel (TXT + USMB) and the triple combination therapy (TXT + USMB + XRT). The data shows the significant cell death associated with the triple combination therapy compared to TXT, TXT + XRT and TXT + USMB. (b) The ratio of TUNEL assay of the six conditions are shown as mean ± SD.

The TUNEL stained images are shown in Figure 8a and the corresponding analysis in Figure 8b. The application of USMB significantly enhanced apoptosis in TXT + USMB compared to TXT alone and in TXT + USMB + XRT compared to TXT + XRT. Under the ultrasound exposure conditions of this study, USMB alone did not statistically increase apoptosis compared to untreated control (Control No USMB), whereas all the other treatment conditions are statistically significant compared to untreated control. In addition, XRT statistically enhanced cell death when combined with TXT.

## 4Discussion

This study demonstrated the potential of combining three independent treatment modalities, specifically, the triple combination of chemotherapy, ultrasound, and microbubbles, and radiotherapy, to enhance the efficacy of clonogenically killing *in vitro* prostate cancer cells. The triple combination therapy is effective under relatively low chemotherapy concentration and ionizing radiation dose, and as the application of ultrasound-and-microbubbles can selectively be focused on the tumour tissue, supports the potential of localizing the treatment to cancerous tissues and enhancing therapeutic efficacy in *in vivo* applications. The focus of this study was on assessing the clonogenic viability of the various double and triple combination therapies under varying chemotherapy and microbubble doses and identifying conditions of synergism.

### 4.1Double combination therapy

#### 4.1.1TXT + XRT

In this study, PC3 clonogenic cell viability decreased with increasing TXT concentration and treatment duration, as expected [20, 22, 35]. Docetaxel, due to its hydrophobic properties, is capable of passively diffusing across cell membranes and as such it is more effective at higher TXT concentrations and longer exposure duration [36]. The maximum cell death was achieved at the highest concentration and longest duration used in this study (TXT at 0.1 nM and 2 h; clonogenic viability ∼75%). Docetaxel, when utilized as a radiosensitizer, although produced significantly lower cell viability when combined with 2 Gy radiotherapy dose, induced an additive effect with TXT at the concentrations and durations investigated. Previous studies at comparable conditions showed similar results [4]. At higher docetaxel drug concentration and radiotherapy dose, the combined treatment of docetaxel and radiotherapy was shown to be synergistic [4]. Docetaxel in combination with 4–6 Gy ionizing radiation, induced a synergistic effect on death of MCF-7 breast cancer cells compared to single treatment [4]. This suggests that synergism in TXT + XRT therapy partly depends on chemotherapy and radiotherapy doses. The enhanced efficacy of double combination therapy of TXT and XRT may be associated with the TXT causing a cell cycle arrest in the radiosensitive G2/M phase of the cell cycle and enhanced apoptosis related to ceramide production [36, 37].

#### 4.1.2TXT + USMB

The double combination of TXT and USMB therapy statistically reduced clonogenic cell viability compared to TXT alone while inducing an additive effect on cell death. The effectiveness of the TXT + USMB therapy depended on both chemotherapy dose and microbubble concentration. The additive bioeffect by the TXT + USMB condition appears to be associated to the lower TXT concentrations (<0.1 nM) used in this study. The ultrasound and microbubble exposure parameters has been shown to enhance cell membrane permeability and enhanced therapeutic efficacy [33, 38, 39]. The enhanced cell death is partly associated with enhanced intracellular uptake of TXT molecules through cell membrane disruption and enhanced endocytosis [40, 41]. The effectiveness of the double combination therapy of TXT and USMB depended on microbubble concentration. It has also been shown that the efficacy of drug delivery depended on the proximity of microbubbles to the plasma membrane and the bubble-to-cell ratio [42, 43, 44]. In addition, the *in vivo* application of USMB at higher ultrasound pressures (1.65 MPa) with docetaxel demonstrated a significant effect on tumour cell death [24].

#### 4.1.3USMB + XRT

Under the conditions of this study, the combined treatment of USMB and XRT is synergistic only under higher microbubble concentrations. Synergism was observed at MB concentrations above 1.0% v/v (volume concentration) when combined with 2 Gy radiation dose. This agrees with the synergistic effect observed in *in vitro* and *in vivo* studies [17, 45, 46, 47, 48]. It was shown that USMB synergistically enhanced the killing of cancer cells through biomechanical perturbation of biological membranes both in *in vitro* and *in vivo* tumour models. The USMB-mediated bioeffects are associated with cavitation induced mechanical stresses on the PC3 cells [15, 24]. USMB-mediated enhancement of ceramide in combination with radiotherapy increases the ceramide level resulting in cell death [46].

#### 4.1.4Triple combination therapy: TXT + USMB + XRT

The triple combination therapy (TXT + USMB + XRT) had a significant effect on cell viability compared to single and double combination treatments. The study demonstrates that under treatment conditions where a single treatment was not effective in killing cancer cells (viability ∼60–80%), the triple combination therapy was highly effective at significantly reducing clonogenic cell viability (∼2%). Docetaxel is associated with various acute and chronic side effects, which are dose dependent [22] and as such the triple combination therapy of TXT + USMB + XRT may reduce toxicity associated with docetaxel while simultaneously enhancing treatment effectiveness. In addition, at all the exposure parameters of TXT concentrations and durations and MB concentrations, the combined treatment of TXT + USMB + XRT is synergistically indicating the potential of optimizing the triple combination treatment for personalized therapy. The underlying bioeffects triggered irreversible biological changes enhancing cell death under chemotherapy and radiotherapy conditions with relatively low cell death. The enhanced efficacy of the triple combination therapy can be associated with various bioeffects including chemosensitization and radiosensitization by USMB, as well as enhanced cell death when treated with both chemotherapy and radiotherapy. Docetaxel, in addition to cytotoxicity and initiation of apoptosis through microtubule stabilization and impairing mitosis, can alter several genes involved in cell survival and oncogenesis [49, 50], which may be associated with the molecular mechanisms by which the triple combination therapy affects the prostate cancer cells. Therapeutic ultrasound with microbubbles can induce a range of cellular bioeffects such as reversible and irreversible sonoporation, endocytosis, exocytosis, calcium influx, apoptosis, autophagy, necrosis, microtubule disruption [12, 15, 51, 52, 53, 54, 55, 56, 57]. The effect of ionizing radiation on cellular DNA is well known through direct and indirect mechanisms, and nanotechnology has been utilized in enhancing radiotherapy [2, 3, 58, 59]. The application of USMB in combination with TXT and TXT + XRT significantly enhances tumour cell death in *in vivo* PC3 model, which agrees with the *in vitro* observations. The beneficial therapeutic effects of ultrasound and microbubbles have been shown with chemotherapy [60, 61] and radiotherapy [32]. The cellular responses from each therapy are complex and further studies are required to understand the overlapping impacts from each therapy.

## 5Conclusions

The triple combination therapy comprised of docetaxel, ultrasound and microbubbles, and ionizing radiation is synergistic in killing PC3 cells *in vitro*. The synergistic effects depended on chemotherapy dose and microbubble concentration. Cell viability decreased by ∼28, ∼37 and ∼38 folds with the triple combination therapy compared to XRT, USMB, and TXT treatments alone, and ∼29, ∼19, and ∼11 folds compared to TXT + USMB, TXT + XRT, and USMB + XRT. Under the exposure conditions of this study, an additive effect was achieved with TXT + USMB and TXT + XRT, whereas with USMB + XRT, synergism was achieved at MB concentrations higher than 1% v/v. This study indicates that the triple combination therapy comprised of docetaxel, USMB and radiotherapy can significantly enhance the desired bioeffects in killing cancer cells while potentially minimizing toxic side effects by utilizing low chemotherapy and radiotherapy doses. This study demonstrated using both *in vitro* and *in vivo* prostate cancer models that the triple combination therapy comprised of docetaxel, USMB and radiotherapy can significantly enhance the desired bioeffects in killing cancer cells while potentially minimizing toxic side effects by utilizing low chemotherapy and radiotherapy doses.

## Declarations

### Author contribution statement

Firas Almasri; Raffi Karshafian: Conceived and designed the experiments; Performed the experiments; Analyzed and interpreted the data; Contributed reagents, materials, analysis tools or data; Wrote the paper.

### Funding statement

This work was supported by Discovery Grants from the Natural Sciences and Engineering Research Council to R.K. (RGPIN-201505941).

### Data availability statement

Data will be made available on request.

### Declaration of interest's statement

The authors declare no conflict of interest.

### Additional information

No additional information is available for this paper.
